# Molecular characterization and phylogenetic analysis of cryptosporidium spp. in pediatric acute gastroenteritis: Epidemiological insights from northeastern Iran

**DOI:** 10.1016/j.nmni.2025.101622

**Published:** 2025-08-23

**Authors:** Bibi Razieh Hosseini Farash, Seyed Aliakbar Shamsian, Fariba Berenji, Mohammad Javad Najafzadeh, Mehdi Zarean, Behrouz Mahmoudi Gorgi, Sahar Soleimanian, Elnaz Nakhaei, Lida Jarahi

**Affiliations:** a Department of Parasitology and Mycology School of Medicine, Mashhad University of Medical Science, Iran; b Cutaneous Leishmania Research Center, Mashhad University of Medical Sciences, Iran; c Mashhad University of Medical Science, Iran; d Department of Community Medicine, Mashhad University of Medical Sciences, Mashhad, Iran

**Keywords:** Cryptosporidium spp., Acute gastroenteritis, Molecular diagnostics, Phylogenetic analysis, Pediatric infections, Northeastern Iran

## Abstract

**Background:**

*Cryptosporidium* spp. are significant zoonotic pathogens causing gastroenteritis, particularly in pediatric populations. This study aimed to investigate the prevalence, molecular characterization, and phylogenetic analysis of *Cryptosporidium* species among children with acute gastroenteritis in northeastern Iran.

**Methods:**

A cross-sectional study was conducted on 138 children aged 3 months to 12 years at Dr. Sheikh Hospital, Mashhad, between January 2023 and June 2024. Stool samples were examined microscopically using Ziehl-Neelsen staining, and molecular analysis was performed targeting the 18S rRNA gene through PCR. Positive samples were sequenced, and phylogenetic trees were constructed to assess genetic diversity.

**Results:**

Microscopic examination detected *Cryptosporidium* oocysts in 23.2 % of samples, while molecular analysis identified *Cryptosporidium* DNA in 26.8 %, demonstrating the superior sensitivity of PCR. Sequencing results revealed *Cryptosporidium parvum* as the predominant species (14/15 samples) with one *Cryptosporidium hominis* case. Phylogenetic analysis confirmed genetic diversity among isolates, highlighting potential zoonotic and environmental transmission routes. No significant associations were observed between infection prevalence and demographic factors such as age or gender (p > 0.05).

**Conclusion:**

This study underscores the importance of molecular diagnostics in accurately identifying *Cryptosporidium* species and understanding their epidemiological significance. The findings contribute to regional knowledge on *Cryptosporidium* infections and highlight the need for targeted public health interventions to reduce disease burden in children.

## 1 Introduction

*Cryptosporidium* species are obligate intracellular protozoan parasites that cause cryptosporidiosis, a gastrointestinal disease of significant concern, particularly in children under five years of age [1]. This parasitic infection poses a major global public health challenge, especially in developing countries, where poor sanitation and limited access to clean water exacerbate its impact. The clinical features of cryptosporidiosis typically include acute diarrhea, abdominal cramps, nausea, and, in more severe cases, dehydration and malnutrition [2,3]. In young children, these manifestations can lead to prolonged illness, growth retardation, and even mortality if untreated [4].

Accurate detection and species-level identification of *Cryptosporidium* spp. are critical for elucidating the epidemiology and transmission routes of the parasite. Conventional diagnostic tools, such as light microscopy of stool samples, often lack the sensitivity and specificity to differentiate among species and genotypes of *Cryptosporidium* [5,6]. These limitations underscore the value of molecular methods—such as polymerase chain reaction (PCR) and DNA sequencing—which offer greater precision, sensitivity, and the ability to identify zoonotic subtypes [7].

Several species of *Cryptosporidium*, notably *C. parvum*, are zoonotic and capable of infecting both humans and a wide range of animals, including livestock, rodents, and pets [8]. This zoonotic potential significantly complicates control strategies, as animal reservoirs may contribute to persistent transmission cycles, especially in rural or peri-urban communities where human-animal interactions are frequent [9].

Molecular characterization enhances our understanding of the parasite's genetic diversity and helps trace its transmission sources. In settings such as Mashhad, Iran, where pediatric gastrointestinal infections are common, comprehensive molecular investigations are essential to map the prevalence and diversity of *Cryptosporidium* spp. in affected populations [10,11]. Identifying circulating species and genotypes provides key insights that can inform treatment strategies, support outbreak surveillance, and guide public health policies. Moreover, such studies can alert health authorities to the emergence of potentially more virulent or drug-resistant strains [12].

This study aims to investigate the molecular characteristics of *Cryptosporidium* spp. in children with acute gastroenteritis attending Dr. Sheikh Hospital in Mashhad. By applying advanced molecular diagnostics, we seek to identify the species and genotypes involved and evaluate their epidemiological relevance. The results are anticipated to inform evidence-based strategies for the prevention and control of cryptosporidiosis, ultimately reducing the disease burden in vulnerable pediatric populations.

## 2 Materials and methods

### 2.1 Study design and population

This descriptive-analytical cross-sectional study was conducted at Dr. Sheikh Hospital in Mashhad between January 2023 and June 2024. A total of 138 pediatric patients aged 3 months to 12 years, clinically diagnosed with acute gastroenteritis and presenting symptoms such as diarrhea, abdominal pain, or signs of dehydration, were enrolled. Inclusion criteria required the presence of written informed consent from the parents or legal guardians. Children with chronic gastrointestinal diseases, such as inflammatory bowel disease, those with laboratory-confirmed concurrent bacterial or viral infections, and immunocompromised patients—including those undergoing chemotherapy or diagnosed with immunodeficiency disorders—were excluded. Demographic and clinical information including age, gender, symptom duration, and hospitalization status was recorded upon enrollment to ensure representation across different subgroups and allow epidemiological correlations.

### 2.2 Ethical approval statement

The study adhered to the ethical standards outlined in the Declaration of Helsinki (1964) and its subsequent revisions, and received approval from the Ethics Committee of Mashhad University of Medical Sciences (IR.MUMS.MEDICAL.REC.1397.659). Written informed consent was obtained from the parents or legal guardians of all participants after detailed explanations of the study objectives and procedures. Participation was entirely voluntary, and parents were assured of their right to withdraw their child from the study at any stage without compromising the quality of their medical care.

### 2.3 Sample collection and preparation

Fresh stool samples were collected in sterile, leak-proof containers, transported on ice, and processed within 2 h of collection. Each sample was divided into two portions. The first portion underwent microscopic examination using modified Ziehl-Neelsen staining to detect *Cryptosporidium* oocysts based on their acid-fast characteristics. The second portion was stored at −20 °C for molecular analysis. Genomic DNA extraction was performed using the QIAamp DNA Stool Mini Kit (Qiagen, Germany), following a protocol modified to enhance DNA yield and purity. These modifications included extending the lysis step to 15 min at 70 °C and incorporating an additional wash step with Buffer AW2 to reduce PCR inhibitors commonly present in stool specimens. These protocol adjustments were validated through preliminary optimization tests to ensure reliable DNA amplification. However, mechanical pretreatment methods such as bead-beating, which could have further improved the disruption of *Cryptosporidium* oocyst walls, were not employed [13]. DNA extracts were quantified using a NanoDrop spectrophotometer and stored at −20 °C until PCR analysis.

### 2.4 Molecular analysis

Conventional polymerase chain reaction (PCR) was utilized to amplify a 347-base pair (bp) fragment of the small subunit ribosomal RNA gene (18S rRNA or SSU-rRNA), which is highly conserved among *Cryptosporidium* species. The primers were designed using the Primer-BLAST tool (NCBI) to ensure high specificity and coverage for clinically relevant species, including *C. parvum*, *C. hominis*, and others. The forward primer (Crypto-F: 5′-GGTGACTCATAATAACTTTACGG-3′) and reverse primer (Crypto-R: 5′-CGCTATTGGAGCTGGAATTAC-3′) were synthesized commercially. The PCR reaction was prepared in a final volume of 25 μL containing 10 μL of 2 × PCR master mix (Pars Tous, Mashhad, Iran), 2 μL of primer mix (10 pmol/μL each), 3 μL of extracted DNA template, and 10 μL of nuclease-free distilled water. Amplification was performed in a thermal cycler (Eppendorf Mastercycler) under the following conditions: initial denaturation at 94 °C for 5 min, followed by 35 cycles of denaturation at 94 °C for 45 s, annealing at 58 °C for 30 s, and extension at 72 °C for 45 s, with a final extension step at 72 °C for 7 min. PCR products were separated by electrophoresis on a 1.5 % agarose gel stained with ethidium bromide and visualized under ultraviolet illumination using a Gel Doc EZ imager (Bio-Rad, USA).

### 2.5 Sequencing and phylogenetic analysis

To evaluate the genetic diversity of *Cryptosporidium* species detected in the study population, PCR amplicons from 15 randomly selected positive samples were subjected to Sanger sequencing at Pishgam Biotech Company (Tehran, Iran). The selected samples represented diverse age groups and both genders to ensure representation of the broader pediatric population. Sequencing was performed in one direction, and chromatograms were manually edited and aligned using BioEdit software. The resulting sequences were compared against reference sequences in the NCBI GenBank database using the BLASTn algorithm to determine species identity.

Phylogenetic relationships among *Cryptosporidium* isolates were analyzed using MEGA11 software. A neighbor-joining (NJ) tree was constructed employing the Kimura 2-parameter model to calculate evolutionary distances. The reliability of the branching pattern was assessed by performing 1000 bootstrap replicates, and values exceeding 70 % were considered statistically robust. The phylogenetic tree retained both topology and branch length information, enabling clear visualization of genetic divergence among isolates. The final tree was edited and annotated for clarity and presentation. All generated nucleotide sequences were submitted to the NCBI GenBank database, and their corresponding accession numbers are provided in the Results section to ensure data transparency and reproducibility.

### 2.6 Statistical analysis

All statistical analyses were performed using SPSS version 16.0 (IBM Corp., Armonk, NY, USA). Descriptive statistics such as medians and interquartile ranges (IQR) were calculated for continuous variables, while frequencies and percentages were used for categorical variables. The Chi-square (χ^2^) test was employed to assess associations between categorical variables, and the Mann–Whitney *U* test was used for non-normally distributed continuous variables. A two-sided p-value of less than 0.05 was considered statistically significant.

## 3 Results

### 3.1 Demographic characteristics

A total of 138 pediatric patients with acute gastroenteritis were enrolled in the study, including 72 males (52.2 %) and 66 females (47.8 %). Participants ranged in age from 3 months to 12 years, with a mean age of 3.65 ± 2.79 years. The highest frequency was observed in the 2–4-year age group, indicating increased susceptibility to gastrointestinal infections in this age range.

#### 3.1.1 Visualization of PCR results

PCR amplification targeting the 18S rRNA gene of *Cryptosporidium* spp. yielded distinct 347 bp bands in positive samples. The presence of these bands, as visualized through gel electrophoresis, confirmed the successful and accurate application of the molecular diagnostic method employed in this study.

### 3.2 Prevalence of cryptosporidium

**Fig. 1:**
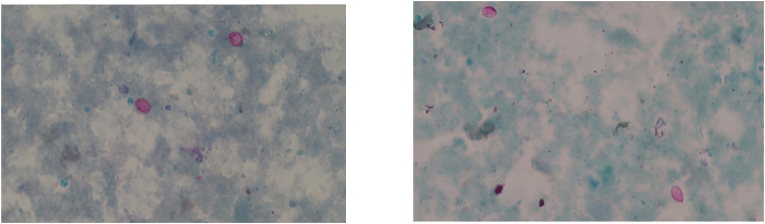
Microscopic visualization of *Cryptosporidium* oocysts stained by modified Ziehl-Neelsen technique (1000 × magnification).

Microscopic analysis using the modified Ziehl-Neelsen staining method detected Cryptosporidium oocysts in 32 of the 138 stool samples, corresponding to a prevalence of 23.2 % (Fig. 1). In contrast, conventional PCR detected Cryptosporidium DNA in 37 samples (26.8 %). The increased detection rate by PCR compared to microscopy was statistically significant (p = 0.021), indicating higher sensitivity of the molecular technique. Among the 37 PCR-positive samples, 32 were also identified by microscopy, while 5 samples were exclusively detected by PCR, further supporting its diagnostic advantage.

### 3.3 Association with demographic factors

Analysis of associations between *Cryptosporidium* infection and demographic factors showed no statistically significant difference between genders (p = 0.529). The prevalence of infection was 26.4 % (19/72) in males and 27.3 % (18/66) in females. The mean age of infected children was 5.0 years, while that of uninfected children was 6.1 years; however, this difference was not statistically significant (p = 0.892). These findings suggest that neither age nor gender significantly influenced the likelihood of *Cryptosporidium* infection in this study population (Table 1).

**Table 1:** Comparison of cryptosporidium detection by gender, age, and diagnostic method in children with acute gastroenteritis.

Variables	Positive Cases (n, %)	Negative Cases (n, %)	Total (n, %)	P-value	Odds Ratio (95 % CI)
Gender (M)	19 (26.4 %)	53 (73.6 %)	72 (100 %)	0.529	0.96 (0.46–2.00)
Gender (F)	18 (27.3 %)	48 (72.7 %)	66 (100 %)		Reference
Microscopy	32 (23.2 %)	106 (76.8 %)	138 (100 %)	0.021	Reference
PCR	37 (26.8 %)	101 (73.2 %)	138 (100 %)		1.21 (0.70–2.09)
Age (Mean)	5 years	6.1 years		0.892	Not applicable

### 3.4 Species identification and phylogenetic analysis

**Fig. 2:**
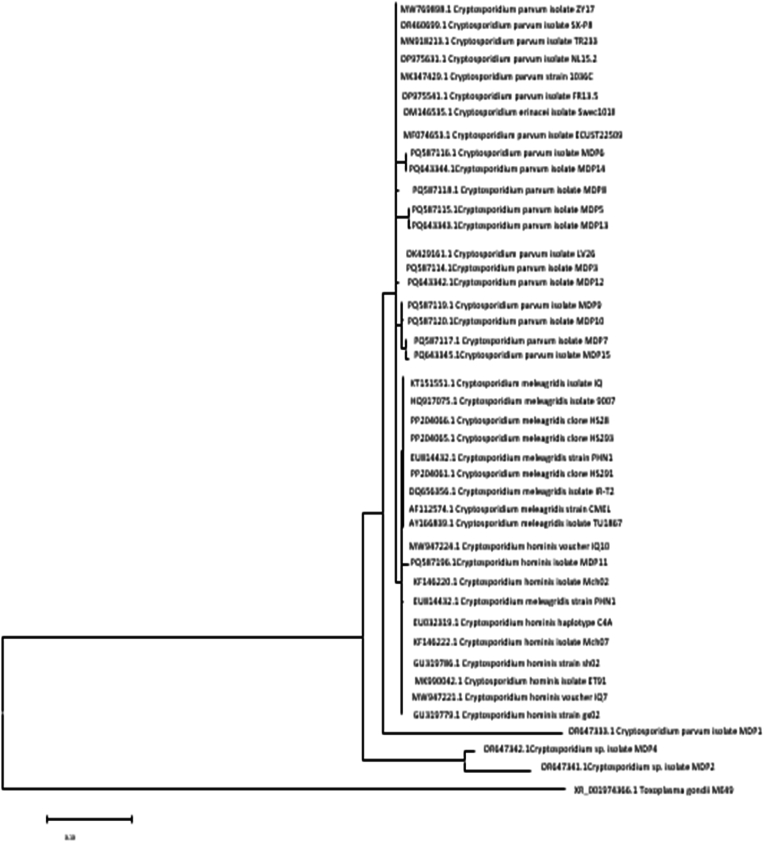
Genetic diversity and phylogenetic analysis of Cryptosporidium parvum and Cryptosporidium hominis strains in children under 12 Years old from Mashhad.

Sequencing analysis was performed on 15 randomly selected PCR-positive samples. Of these, 14 samples were identified as *C*. *parvum* and one as *C*. *hominis*, showing 99–100 % similarity to reference sequences in the NCBI BLAST database. The selection was based on demographic diversity. Due to resource limitations, the remaining PCR-positive samples were not sequenced. Accession numbers for the submitted sequences are as follows: *C. parvum* – PQ643342, PQ643343, PQ643344, PQ643345, PQ587114, PQ587115, PQ587116, PQ587117, PQ587118, PQ587119, PQ587120, OR647333, OR647342, OR647341; *C. hominis* – PQ587196.1. (Fig. 2).

The phylogenetic tree was constructed in MEGA11 using the Maximum Likelihood method based on the Tamura-Nei model (Fig. 2). The analysis showed that most of the study sequences—specifically PQ643342 to PQ643345 and PQ587114 to PQ587120—clustered tightly within a well-supported monophyletic group corresponding to *C*. *parvum*. These sequences exhibited short branch lengths and minimal variability, indicating a genetically conserved strain during the initial phase of sample collection.

In contrast, three sequences OR647333, OR647342, and OR647341 were positioned outside the main *C. parvum* cluster, forming separate sub-branches. These isolates were obtained approximately one year prior to the initial batch of samples. Their distinct placement may reflect temporal genetic divergence within circulating *C. parvum* strains in the region, potentially driven by ecological or host-related changes over time.

Additionally, the *C*. *hominis* isolate MDP11 (accession no. PQ587196.1) grouped into a clearly distinct clade, confirming interspecies divergence and further validating the specificity of the molecular identification process.

The evolutionary history was inferred using the Maximum Likelihood method with the Tamura-Nei model [14]. The tree with the highest log likelihood (−1517.97) is shown. Initial tree(s) for the heuristic search were obtained automatically by applying Neighbor-Join and BioNJ algorithms to a matrix of pairwise distances estimated using the Tamura-Nei model, and then selecting the topology with superior log likelihood value. The tree is drawn to scale, with branch lengths measured in the number of substitutions per site. This analysis involved 43 nucleotide sequences. There were a total of 320 positions in the final dataset. Evolutionary analyses were conducted in MEGA11 [15]. *Toxoplasma gondii* was used as an outgroup.

## 4 Discussion

This study provides valuable insights into the molecular epidemiology of *Cryptosporidium* spp. among children with acute gastroenteritis in Mashhad, Iran, revealing a notably high prevalence of *C. parvum* (26.8 % by PCR). The detection rate observed through PCR was significantly higher than that of conventional microscopy (23.2 %), reaffirming the known limitations of microscopic methods in identifying low oocyst counts or distinguishing between *Cryptosporidium* species. These findings are consistent with studies by Khurana et al. and Ryan et al., which highlighted PCR's superior sensitivity and its ability to detect mixed or subclinical infections that microscopy may overlook [16,17]. Integrating molecular diagnostics into routine clinical and public health settings is therefore essential for accurate diagnosis, early intervention, and the prevention of disease spread, especially in vulnerable pediatric populations.

The predominance of *C. parvum* over *C. hominis* in this study is particularly notable given the urban setting of Mashhad and the limited direct contact between the study population and livestock. This suggests that zoonotic transmission in this context may occur through indirect environmental pathways, such as contaminated water, fresh produce, or public surfaces, rather than direct animal contact. Similar patterns have been reported in urban regions of Egypt and Turkey, where *C. parvum* dominated among children despite minimal animal exposure [7,16]. These findings underscore the zoonotic potential of *C. parvum*, which remains a significant public health concern even in urbanized areas. The importance of zoonotic transmission in the context of urban infrastructure highlights the need for One Health approaches, emphasizing the interconnectedness of human, animal, and environmental health systems.

Phylogenetic analysis revealed that all *C. parvum* isolates clustered within a monophyletic group, yet showed minor sub-branching patterns, indicating some degree of intra-species genetic variability. These findings are in line with reports by Garcia-R et al., who demonstrated that *Cryptosporidium* populations are often heterogeneous within a single geographical region [18]. This subtle genetic variability within the main *C. parvum* cluster (isolates PQ643342–PQ643345 and PQ587114–PQ587120) suggests a dominant strain with minor variations that may influence transmission dynamics or pathogenicity. For instance, these variations could reflect adaptations to environmental pressures or differences in host susceptibility, warranting further investigation into their epidemiological significance.

The separation of three older *C. parvum* isolates (*OR647333, OR647342*, *OR647341*), collected approximately one year prior to the main batch, from the primary *C. parvum* cluster suggests temporal genetic divergence. **Notably, these isolates were obtained during the COVID-19 pandemic, a period marked by disruptions in sanitation services, increased household crowding, and altered hygiene practices, which may have influenced the selection or persistence of distinct** *C. parvum* **strains** [28]**. In contrast, the post-pandemic isolates formed a tighter monophyletic group, potentially indicating the re-emergence of a dominant strain following the relaxation of pandemic-related restrictions.** This divergence could result from ecological changes, such as alterations in water sources or host contact patterns, or selective pressures, such as therapeutic interventions or host immune responses. The predominance of *C. parvum* in the analyzed samples (14 out of 15) highlights the likely role of zoonotic sources, such as livestock or contaminated water, in the transmission of cryptosporidiosis in the study region. A similar study in Southwestern Ontario, Canada, demonstrated that bovine C. parvum cases were associated with a three-fold increase in human cryptosporidiosis risk, with environmental factors like temperature and water contamination playing a significant role in zoonotic transmission [19].These findings underscore the need for enhanced surveillance of water sources and improved livestock management practices to mitigate transmission risks, **particularly during public health crises that may exacerbate environmental contamination.**

The identification of a single *C. hominis* isolate (PQ587196.1) in a distinct clade confirms interspecies divergence and validates the accuracy of the molecular methods employed. This observation emphasizes the importance of public health measures, such as water treatment and hygiene education, to prevent human-to-human transmission of *C. hominis*.

The observed diversity may reflect seasonal and temporal factors, such as fluctuations in temperature, precipitation, or intermittent contamination of municipal water sources. It is also possible that genetic diversity is influenced by human and animal movement, variability in sanitation infrastructure, and episodic exposure to contaminated food or recreational water. The single *C. hominis* isolate formed a separate and distinct clade, confirming the evolutionary divergence between these two species. This distinction aligns with prior studies noting *C. hominis* as primarily anthroponotic and *C. parvum* as zoonotic, with differing epidemiological patterns and public health implications [20].

The relatively high infection rate of *C. parvum* observed in our study may also be shaped by environmental risk factors, including suboptimal sanitation infrastructure, aging water distribution systems, limited wastewater treatment, and inadequate public hygiene practices. Such conditions can facilitate the persistence and transmission of *Cryptosporidium* oocysts, which are resistant to standard chlorination procedures. A study by Nhambirre et al. in Mozambique similarly identified poor sanitation and environmental exposure as major contributors to pediatric *Cryptosporidium* infections [21]. This reinforces the urgency of addressing infrastructural and behavioral determinants of transmission, particularly in urban populations where close contact and shared facilities can amplify spread.

When comparing our findings with those of other Iranian regions, notable geographic variability in species distribution emerges. For example, Berahmat et al. (2017) reported *C. parvum* as the dominant species in multiple provinces, while Izadi et al. (2020) identified a higher prevalence of *C. hominis* among immunocompromised individuals in central Iran [22,23]. These differences may stem from variations in host immune status, water source contamination, and localized hygiene practices. They also highlight the need for region-specific surveillance strategies that consider socio-demographic and environmental variables influencing transmission dynamics.

Despite the strengths of this study including a robust sampling frame, molecular confirmation, and phylogenetic characterization certain limitations should be acknowledged. The use of conventional endpoint PCR, although effective for sequencing, lacks the quantitative precision and higher sensitivity offered by real-time PCR (qPCR), which has been recommended for routine diagnostics and outbreak investigations [[24], [25], [26]]. Furthermore, while DNA extraction was enhanced by prolonging the lysis step and adding an additional wash to reduce PCR inhibitors, the absence of mechanical pretreatment (e.g., bead-beating) may have limited the yield of DNA from oocysts, as suggested by previous reports [26]. Another limitation is that only 15 PCR-positive samples were sequenced, selected based on demographic diversity. This sample size may not fully capture the genetic variability of *Cryptosporidium* strains circulating in the study population. **Future studies should prioritize broader sequencing efforts, including whole-genome sequencing and gp60 subtyping, to achieve higher resolution analysis of intra-species variation and elucidate the impact of external factors, such as pandemics, on strain dynamics** [27].

Moreover, it is important to consider that the COVID-19 pandemic, which overlapped with the study period, may have affected transmission patterns and healthcare-seeking behaviors. **The distinct clustering of the three** *C. parvum* **isolates collected during this period suggests that pandemic-related factors, such as disruptions in sanitation services, increased household crowding, or changes in hygiene practices, may have driven the selection of unique strains** [28]**. These findings highlight the importance of monitoring zoonotic pathogens during global health crises, as such events can alter the epidemiology of enteric infections.** Disruptions in sanitation services, increased household crowding, and changes in hygiene practices could have influenced the incidence and detection of enteric pathogens during this time.

The importance of timely and accurate diagnosis of cryptosporidiosis, particularly in children, has been emphasized in several major studies. Hunter & Kotloff and Akinnubi et al. have shown that early detection and targeted treatment of *Cryptosporidium* infections can reduce the duration and severity of illness, as well as long-term complications such as growth retardation and malnutrition [[29], [30], [31]]. Therefore, integrating advanced molecular techniques and expanding epidemiological surveillance are critical steps toward effective disease management.

## 5 Conclusion

This study highlights the predominance of *C. parvum* in pediatric cases of acute gastroenteritis in Mashhad, Iran, and underscores the role of indirect zoonotic and environmental transmission in urban settings. The findings demonstrate the value of molecular tools in enhancing diagnostic accuracy and uncovering genetic diversity among circulating strains. **The temporal divergence of** *C. parvum* **isolates collected during the COVID-19 pandemic suggests that global health crises can influence the genetic epidemiology of zoonotic pathogens, warranting heightened surveillance during such periods.** Despite certain methodological constraints, the study establishes a foundation for future research aimed at clarifying transmission dynamics and guiding interventions. Longitudinal studies incorporating qPCR, gp60 subtyping, and whole-genome sequencing, along with optimized DNA extraction protocols (including mechanical disruption), are warranted to provide deeper insights into the genetic and epidemiological dynamics of *Cryptosporidium*. Addressing infrastructural gaps in water sanitation and hygiene, along with tailored public health strategies, will be essential to reduce the burden of cryptosporidiosis among children in endemic regions. These findings can assist policymakers in designing region-specific public health programs, focusing on enhancing water infrastructure and sanitation practices to mitigate the spread of cryptosporidiosis.

## CRediT authorship contribution statement

**Bibi Razieh Hosseini Farash:** Writing – review & editing, Writing – original draft, Project administration, Methodology, Investigation, Funding acquisition, Formal analysis, Data curation, Conceptualization. **Seyed Aliakbar Shamsian:** Resources. **Fariba Berenji:** Methodology. **Mohammad Javad Najafzadeh:** Software. **Mehdi Zarean:** Validation. **Behrouz Mahmoudi Gorgi:** Investigation. **Sahar Soleimanian:** Investigation. **Elnaz Nakhaei:** Investigation. **Lida Jarahi:** Formal analysis.

## Funding source

This study was generously supported by the Vice Chancellor for Research at 10.13039/501100004748Mashhad University of Medical Sciences under Grant No. 970502.

## Declaration of competing interest

The authors declare the following financial interests/personal relationships which may be considered as potential competing interests:Bibi Razieh Hosseini Farash reports financial support was provided by 10.13039/501100004748Mashhad University of Medical Sciences, Mashhad, Iran. Bibi Razieh Hosseini Farash reports a relationship with Mashhad University of Medical Sciences, Mashhad, Iran. that includes: employment. Bibi razieh Hosseini farash has patent #970502 pending to 970502. We hereby declare that none of the authors have any relationships or activities that could be interpreted as a conflict of interest regarding the content of this manuscript or its submission to New Microbes and New Infections. None of the authors serve in any editorial capacity for this journal or have any financial, personal, or professional affiliations that could influence the objectivity of the research presented If there are other authors, they declare that they have no known competing financial interests or personal relationships that could have appeared to influence the work reported in this paper.
